# A Geometric Morphometric Study on Sexual Dimorphism in Viscerocranium

**DOI:** 10.3390/biology11091333

**Published:** 2022-09-09

**Authors:** Diana Toneva, Silviya Nikolova, Elena Tasheva-Terzieva, Dora Zlatareva, Nikolai Lazarov

**Affiliations:** 1 Department of Anthropology and Anatomy, Institute of Experimental Morphology, Pathology and Anthropology with Museum, Bulgarian Academy of Sciences, 1113 Sofia, Bulgaria; 2 Department of Zoology and Anthropology, Faculty of Biology, Sofia University, 1164 Sofia, Bulgaria; 3 Department of Diagnostic Imaging, Faculty of Medicine, Medical University of Sofia, 1431 Sofia, Bulgaria; 4 Department of Anatomy, Histology and Embryology, Faculty of Medicine, Medical University of Sofia, 1431 Sofia, Bulgaria

**Keywords:** viscerocranium, sexual dimorphism, 3D landmarks, geometric morphometrics, computed tomography

## Abstract

**Simple Summary:**

Sex estimation is a crucial step in the identification of unknown bone remains. The accuracy of sex estimation methods depends on the level of sexual dimorphism manifested by the human bones. Therefore, the evaluation of sex differences of particular bones of the skeleton is an important preceding stage. We have used geometric morphometric techniques to investigate the sexual dimorphism in the size and shape of the facial part of the skull and its subregions. Our results show that the facial skeleton in males and females differs more in size than in shape, so its overall size is a more useful sex indicator than its shape. The same result is observed for all facial subregions studied here. However, the best discrimination between the male and female skulls is achieved when both size and shape are considered together.

**Abstract:**

The level of sexual dimorphism manifested by human bones is an important factor for development of effective sex estimation methods. The aim of the study was to investigate the sexual dimorphism in the size and shape of the viscerocranium using geometric morphometric techniques. It also aimed to explore the sex differences in distinct viscerocranial regions and to establish the most dimorphic region with regard to size and shape. Computed tomography images of 156 males and 184 females were used in the study. Three-dimensional coordinates of 31 landmarks were acquired. Five landmark configurations were constructed from the viscerocranium and its orbital, nasal, maxillary, and zygomatic region. Generalized Procrustes superimposition, principal component analysis, and discriminant analysis were applied to each configuration. The significance of the sex differences in size and shape was assessed and significant differences were found in all configurations. The highest accuracy was obtained from both shape and size of the whole viscerocranium. Based on size only, the highest accuracy was achieved by the nasal region. The accuracy based on shape was generally low for all configurations, but the highest result was attained by the orbital region. Hence, size is a better sex discriminator than shape.

## 1. Introduction

Sex estimation methods have been developed for different bones and bone structures. The cranium has been widely studied for this purpose, although the pelvis and long bones have demonstrated more pronounced sexual dimorphism [1]. The cranial sexual dimorphism is mainly manifested in the larger size and greater overall robusticity in males compared to those in females. The sex differences in the human cranium are determined by various factors such as genetic, environmental, socioeconomic, etc. [2,3,4,5]. Thus, the sex estimation appears a complex task since the differences between male and female crania are not solely dependent on a single systemic influence such as hormone levels [6]. Most of the sex differences in cranial size and shape are interrelated with sex differences in physiological and functional parameters characterizing the musculoskeletal and metabolic system such as muscle mass, muscle strength, ventilation volume, volume of oxygen intake, etc. Due to the size differences, males require larger muscles and respectively larger insertion sites to support and move the head [7]. Besides, males develop both absolutely and relatively larger midfacial dimensions than females in connection with the larger airway passages in respect with energy expenditure and oxygen consumption [8,9]. The climatic conditions and latitude have also been associated with cranial shape and especially with facial shape and positioning [10]. Thus, a mixture of various factors modulates the manifestation of sexual dimorphism in different population groups.

Most sexing methods are based on metric characteristics, since they are more reliable compared to the subjectively assessed morphoscopic traits. Yet, there are recent studies which have aimed to quantify traditionally used cranial and mandibular morphoscopic traits [11,12] and could be useful in cases of fragmented skulls. Sex estimation methods have been elaborated on different types of material and by different approaches. Many previous morphometric studies on cranial sexual dimorphism have been carried out on dry bones using calipers [1,13,14,15,16] or digitizer [6,17]. On the other hand, the two-dimensional (2D) and three-dimensional (3D) images generated by different techniques allow direct measuring on the specimens and collection of 2D or 3D coordinates of definite anatomical landmarks. The advantages of the morphometry based on 3D landmark coordinates is the ability to divide the cranium into constituent regions and to maintain at the same time a high number of measurable traits [18], to calculate various types of measurements as well as to differentiate the size and shape components of a particular structure [19]. Data acquired from medical imaging modalities have been widely used in the last years. In this regard, computed tomography (CT) scans have been used in many studies on cranial sexual dimorphism [7,20,21,22,23,24,25,26,27,28,29,30].

Data analysis is another important part in the study of sexual dimorphism. Over the years, the most applied classification method for the development of sex estimation models is discriminant function analysis [1,5,7,13,14,15,16,17,21,23,31,32,33,34,35,36]. Logistic regression analysis has also been commonly applied in sex estimation studies [22,23,24,37,38]. Currently, data mining and machine learning approaches including support vector machines [28,30,39,40,41], artificial neural networks [27,29,41], decision trees [42,43], and classification rule algorithms [43] are extensively applied.

The performance of sex estimation methods heavily depends on the level of sexual dimorphism expressed in the studied population. The development of more effective methods for sex estimation relies on a better understanding of the variables influencing the manifestation of dimorphic traits [6]. According to Sierp and Henneberg [44], sexing methods could be more widely applicable if they are based on characteristics which reflect more straightforward the influence of gonadal steroid hormones on the development of skeletal morphology, irrespective of the size of the individual. However, the size is a substantial component of sexual dimorphism, which is why it could not be fully neglected. In this regard, geometric morphometrics (GM) is a rational analytical approach, which allows separately studying the sex differences in size and shape.

The face is a distinctive region of the human body between males and females [45]. This suggests the presence of sex differences in the underlying bone structure as well. Generally, the viscerocranium surrounds the oral cavity, pharynx, and upper airway passages enclosing the first part of the respiratory and gastrointestinal tracts [46,47]. It houses and protects the sensory organs of olfactory and gustatory systems, and supports the content of the eye sockets. In addition, the facial bones serve as a frame for attachment of the facial soft tissues. Along with facilitating the processes of respiration and mastication, the facial soft tissues are also actively involved in human verbal (speech) and non-verbal (facial gesture) communication. In the human evolution, one of the main changes observed in the skull is shortening of the face [48,49]. This morphological transformation has been attributed to diet and dietary behavior, respiratory/energetic demands, and climate adaptation, as well as to social and cultural factors. Besides, the facial reduction in human evolution is associated with reduction in size of some sexually dimorphic structures, such as canines and supraorbital ridge [49]. Thus, the sex differences in the human cranium are greater in the fossil ancestors compared to the contemporary human populations [2].

The viscerocranium undergoes a longer ontogenetic growth and reaches the adult size later compared to the neurocranium [50], so it grows at a slower rate and for a longer period of time [51]. At birth, the size of the viscerocranium is 76% of its adult size, while that of the neurocranium is above 80% [50]. During growth, the shape and size of the facial skeleton intensively change under the influence of various local and systemic factors [52]. Bastir et al. [53] have established that the growth of the viscerocranium continues until 15.7 years of age, while Noble [50] has found that this process lasts until 18 years of age. The growth of the viscerocranium is related to the development and eruption of the dentition and the development of muscles of mastication [47]. Thus, during ontogeny, the viscerocranium undergoes not only a considerable increase in size, but also a substantial shape modification, becoming relatively higher and more prognathic [50,53].

There have been several GM studies on cranial sexual dimorphism, which consider the shape differences in viscerocranium. However, some of them have discussed the sex differences in viscerocranium as a part of the whole cranium and have not examined its configuration separately [6,20,54,55]. Only a few studies have considered the viscerocranium as a separate entity and tested the discrimination ability of its shape and size [25,56,57,58]. According to these studies, the shape of the upper face has discriminated the male and female crania with comparatively high accuracy: varying from 83% [25,57] to the utmost accuracy of 100% [56], which indicates the extremely good sex estimation potential of its morphological characteristics. Besides, distinct subregions within the viscerocranium have revealed a varying level of sexual dimorphism [56,57,59]. During growth, the orbital region is the first to attain its final size and accordingly it has less time to differentiate between the two sexes, while the structures related to mastication and teeth eruption, such as mandible and maxilla, are the last to grow, and thus they can develop more apparent sex differences under the relatively longer hormonal control [60]. Hence, based on the limited number of previous studies, it could be expected that the viscerocranium would provide high rates of correct sex classification and the strength of sexual dimorphism would differ in its constituent regions. Therefore, the objectives of the present study are: (1) to investigate and further extend the knowledge of sexual dimorphism in size and shape of the viscerocranium using GM techniques; (2) to check the sex discrimination ability of the facial skeleton in the studied population; (3) to explore the sex differences in certain viscerocranial regions (orbital, nasal, maxillary, and zygomatic) in order to establish the most dimorphic region with regard to size and shape considering the time-span of their growth and development.

## 2. Material and Methods

The sample of the present study included 340 CT scans of adult Bulgarians. It consisted of 156 males (mean age: 54.1 ± 17.0 years; 19–89 years) and 184 females (mean age: 57.4 ± 16.0 years; 20–94 years). The scanning was performed with a CT scanner Toshiba *Aquilion 64*. The scanning parameters were as follows: 120 kV tube voltage, tube current in the range 165–500 mA, 0.5 s exposure time, and detector configuration of 32 × 0.5 mm. The image reconstruction protocol included: a reconstruction matrix of 512 × 512 pixels, slice thickness of 0.5 mm, reconstruction interval of 0.3 mm, and convolution kernel FC63. The CT images were collected from 2017 to 2021. The DICOM series were anonymized in advance except the information about the sex and age of the individuals. The sample included only individuals without any pathological alterations of the facial bones. The study was performed after approval by the Human Research Ethics Committee at the Institute of Experimental Morphology, Pathology and Anthropology with Museum, Bulgarian Academy of Sciences.

**Figure 1:**
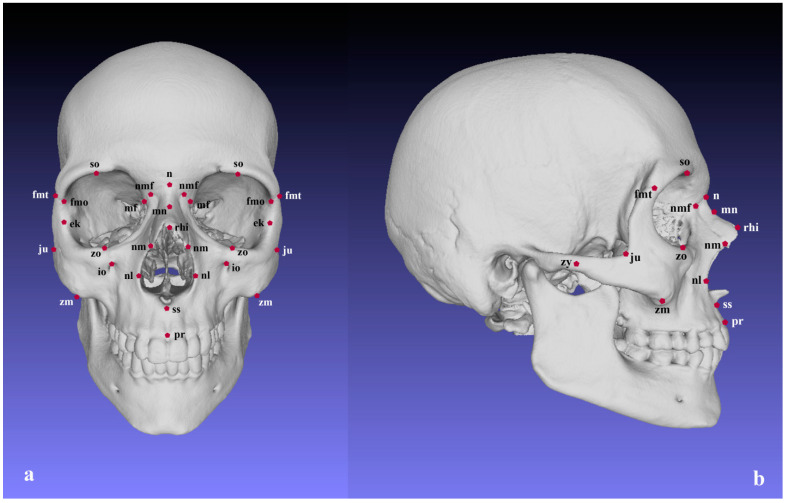
Landmarks of the viscerocranium: (**a**) frontal view; (**b**) lateral view.

The DICOM series were processed in InVesalius (CTI, Brazil) for generation of surface models of the skulls. The predefined threshold for bone tissue (+227 to +3071 HU) was used for segmentation of the bone .stl format. They were used for recording the 3D coordinates of 31 landmarks describing the viscerocranium. Five of the landmarks were located in the mid-sagittal plane and 13 were bilateral (Table 1; Figure 1). The digitization of the landmarks was performed with the “Pick Point” tool in MeshLab [61]. All landmark data were collected by one examiner.

Based on the digitized landmarks, five configurations were arranged: viscerocranium, orbital region, nasal region, maxillary region, and zygomatic region. The landmark data in each configuration were processed using GM techniques. Data for shape (Procrustes coordinates) and size (centroid size, CS) were calculated for each configuration. A generalized Procrustes superimposition (GPS) was applied to the raw landmark coordinates in MorphoJ [65]. This superimposition is a method for aligning shapes using scaling, translation, and rotation. The Procrustes superimposition effectively separates shape and size, but does not remove the covariation between these two components. Consequently, multivariate regression of the Procrustes coordinates (symmetric component) on “ln CS” was applied to test the allometric effect of size on shape. Permutation test (with 10,000 permutations) was performed to test the null hypothesis of independence between size and shape. To avoid the allometric effect, the regression residuals of the studied configurations were used as input data for further analyses of shape.

The sex differences in the size and shape of each configuration were tested for statistical significance. The significance of the sex differences in CS was evaluated by the independent t-test or Mann–Whitney U-test, depending on the results of the normality test and equal variance test. The shape variables of the male and female crania were compared by one-way PERMANOVA, since the test for multivariate normal distribution failed in all configurations. The test for multivariate normal distribution of the shape variables was performed in the online calculator at WebPower [66]. The sex differences in size were evaluated in SPSS (SPSS Inc., Chicago, IL, USA) and the shape differences were tested using PAST [67].

The shape variation in all configurations was studied by principal component analysis (PCA). Variance–covariance matrices were computed based on the regression residuals. The shape variation associated with the first principal component (PC) was visualized in MorphoJ. For that purpose, wireframe graphs were constructed to illustrate the extreme shape configurations along the corresponding PC axis. The scale factor was set at 0.1 Procrustes units.

The classification power of the CS of each landmark configuration was evaluated by univariate discriminant analysis. Multivariate discriminant analysis was performed to evaluate the discrimination ability of the shape variables. It was applied to the PC scores obtained after PCA on the regression residuals of each configuration. The PCs cumulating 95% of the total variance were used in the analysis. In addition, multivariate discriminant analysis was carried out on both the CS and shape variables in order to evaluate the classification accuracy when size and shape were considered together. For this purpose, PCA was carried out (in PAST) on the matrices generated from the regression residuals and “ln CS”. The PCs that accounted for 95% of the total variance were used as input variables in the discriminant analysis. The classification accuracy was calculated using leave-one-out cross-validation. The univariate and multivariate DA were carried out in SPSS.

The intraobserver error was assessed by digitizing 3 times all landmarks on 40 crania. Twenty male and 20 female crania were randomly selected for the purpose. The measurement error of each landmark was evaluated based on its standard deviation. The landmark standard deviation was calculated for each specimen based on the Euclidian distances of the repeated placements of that landmark to the landmark centroid (i.e., the mean x-, y-, and z-coordinates of the landmark obtained from the different trials) [68]. The intraobserver error for each landmark was estimated by averaging the errors across the 40 specimens. The effect of the landmark measurement errors on size and shape of the viscerocranium was evaluated based on the CS values and the first two PCs. The CS values from the different trials were compared with Intraclass Correlation Coefficient (ICC, Absolute Agreement). A scatterplot of the first two PCs was created to visualize the distribution of the different trials of all 40 specimens.

## 3. Results

### 3.1. Intraobserver Error

**Figure 2:**
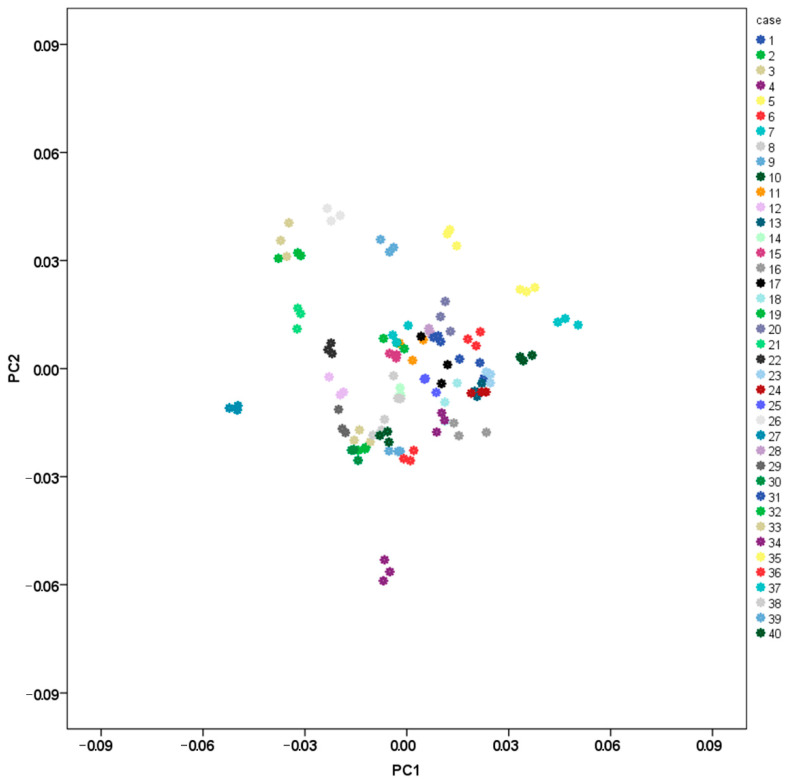
PCA scatterplot: landmark digitization error (three trials of 40 specimens).

The values of the measurement errors were lower than 1 mm for all landmarks (Table S1). The ICC value of the CS (0.997) indicated excellent reliability. The distribution of the different trials along the axes of the first two PCs showed that the trials of each specimen were placed closely together (Figure 2). Accordingly, the effect of the precision error of landmark digitization on size and shape of the viscerocranium was considered as relatively small.

### 3.2. Size

The CS of the viscerocranium was significantly greater in males than in females. The configurations of the separate facial subregions also showed significant sex differences in size (Table 2). The viscerocranium size assigned correctly 81.8% of the male and female crania. The nasal region attained the highest classification accuracy among the facial subregions (Table 3). The maxillary and zygomatic region provided accuracy rates close to that of the nasal region. Thus, the CS of the orbital region yielded the lowest result among all studied configurations.

### 3.3. Allometry

The results of the multivariate regression showed that the influence of the viscerocranium size on the shape was significant (*p* < 0.001) and the size accounted for 3.1% of the total shape variance. Significant dependence of the shape on size was also established in the facial subregions (*p* < 0.001). However, the percentage of the total morphological variation explained by size varied in these configurations (orbital region: 3.9%; nasal region: 4.0%; maxillary region: 1.4%; zygomatic region: 3.7%). As size has a significant effect on shape in all configurations, the tests for sex differences in shape and PCA were conducted on the residuals of the multivariate regressions.

### 3.4. Shape

The male and female crania differed significantly in shape of the viscerocranium (F = 5.02, *p* ≤ 0.001). The facial subregions also demonstrated significant sex differences in their shape: orbital region (F = 9.16, *p* ≤ 0.001), nasal region (F = 6.12, *p* ≤ 0.001), maxillary region (F = 4.33, *p* ≤ 0.001), and zygomatic region (F = 6.76, *p* ≤ 0.001).

**Figure 3:**
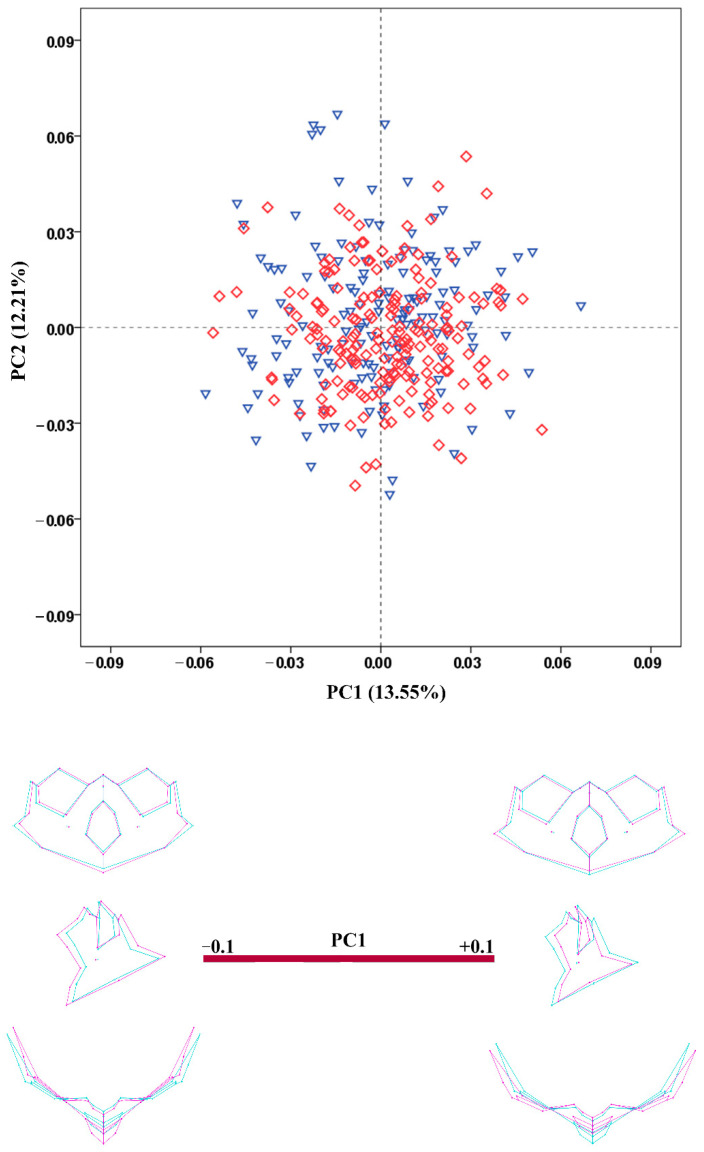
PCA scatterplot for the viscerocranium configuration. The wireframes illustrate the shape variation along the axis of PC1. Male crania—blue triangles; female crania—red rhombs; extreme shape configurations—purple wireframes; mean shape configuration—light blue wireframe.

**Figure 4:**
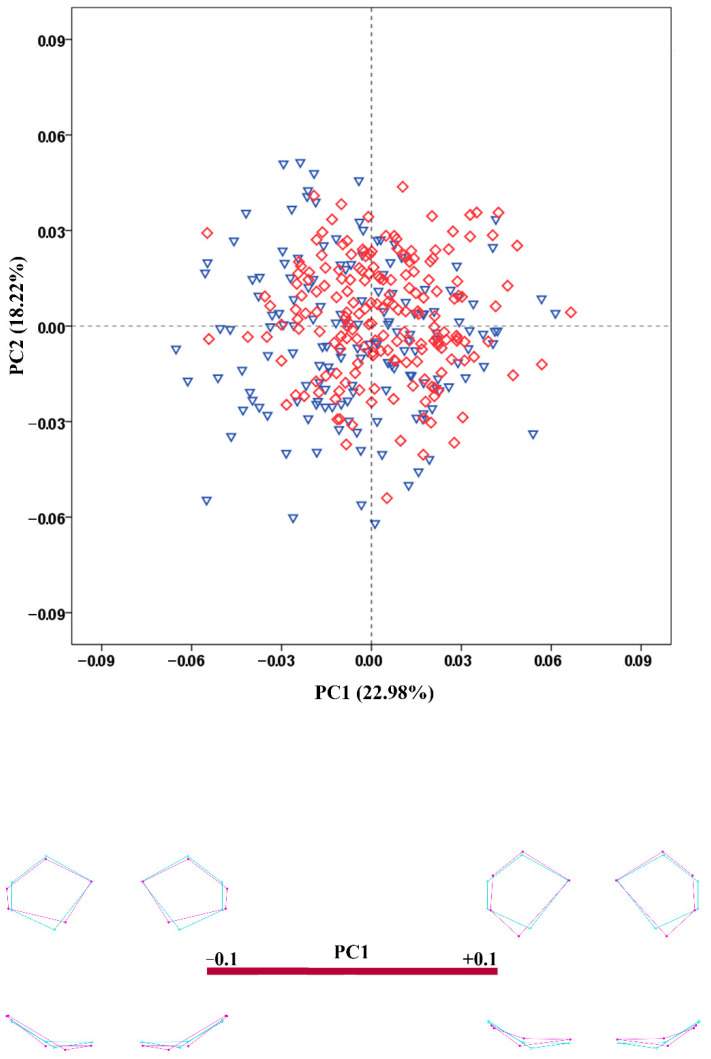
PCA scatterplot for the configuration of the orbital region. The wireframes illustrate the shape variation along the axis of PC1. Male crania—blue triangles; female crania—red rhombs; extreme shape configurations—purple wireframes; mean shape configuration—light blue wireframe.

**Figure 5:**
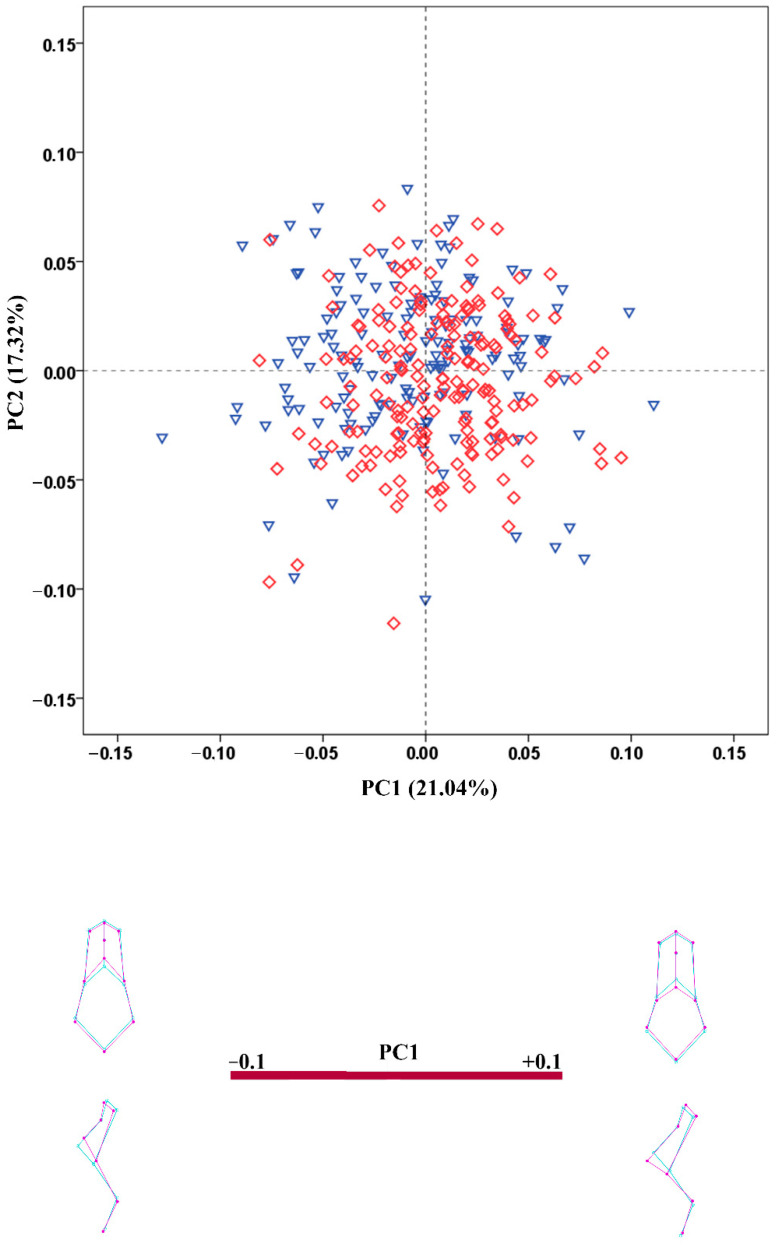
PCA scatterplot for the configuration of the nasal region. The wireframes illustrate the shape variation along the axis of PC1. Male crania—blue triangles; female crania—red rhombs; extreme shape configurations—purple wireframes; mean shape configuration—light blue wireframe.

**Figure 6:**
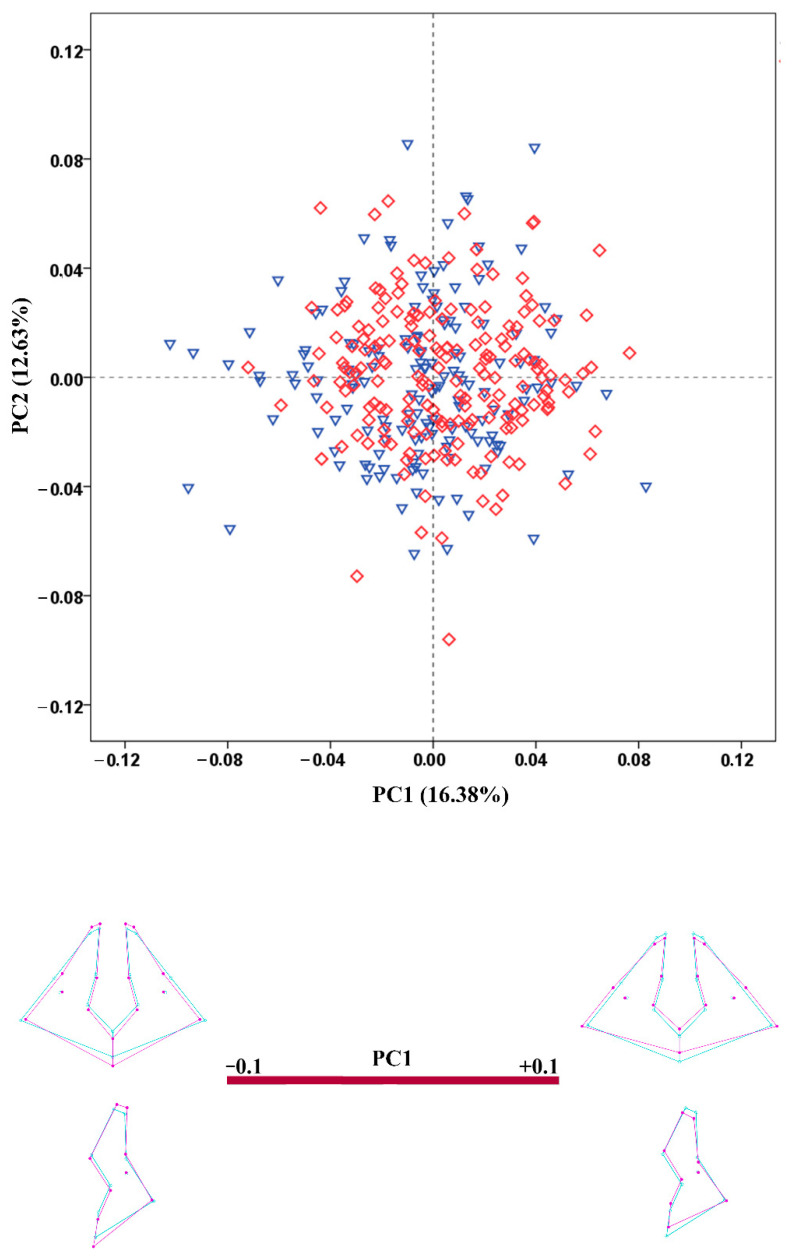
PCA scatterplot for the configuration of the maxillary region. The wireframes illustrate the shape variation along the axis of PC1. Male crania—blue triangles; female crania—red rhombs; extreme shape configurations—purple wireframes; mean shape configuration—light blue wireframe.

**Figure 7:**
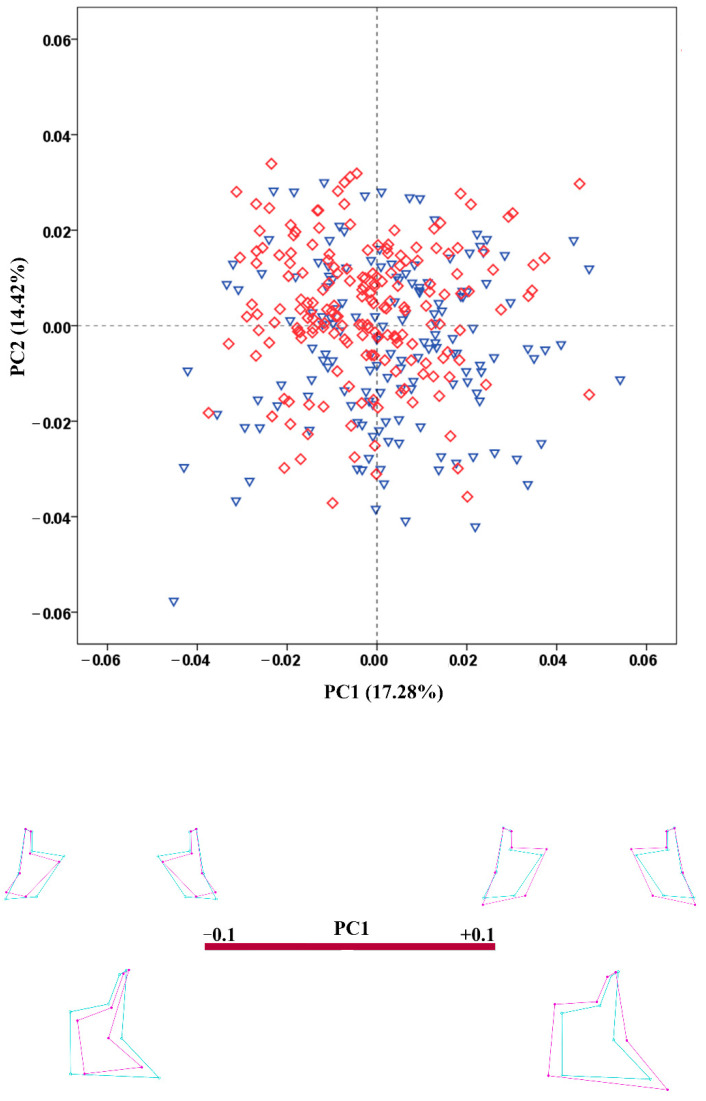
PCA scatterplot for the configuration of the zygomatic region. The wireframes illustrate the shape variation along the axis of PC1. Male crania—blue triangles; female crania—red rhombs; extreme shape configurations—purple wireframes; mean shape configuration—light blue wireframe.

The results of the PCA conducted on the regression residuals showed considerable overlap of the male and female crania along the extracted PCs in all configurations. The percentage variation explained by PC1 and PC2 was very low in all configurations. Figure 3, Figure 4, Figure 5, Figure 6 and Figure 7 demonstrate the scatterplots of the first two PCs of each configuration and the shape transformation observed along the PC1 axis. The first PC accounting for the greatest part of the total shape variance in the viscerocranium was associated with changes in the relative width and length of the facial skeleton (Figure 3). The shape variation described by PC1 of the orbital configuration showed a relocation of the landmarks defining the height and width of the orbits (Figure 4). According to the PCA conducted on the nasal configuration, the greatest variation was observed in the relative length of the nasal bones (Figure 5). The PC1 of the maxillary configuration described shape changes in the relative height and width of this region (Figure 6). In the configuration of the zygomatic bones, the greatest variation in the sample was observed in the proportion between the area of the malar surface and the relative length of the frontal process as well as in the position of the most lateral point of the zygomatic arch (Figure 7). The wireframe graphs demonstrated the shape transformation associated with the greatest shape variation in the sample. Therefore, they should not be confused with illustrations of the sex differences in the shape of the corresponding configuration, since no separation between the two groups was observed on the scatterplots.

The shape of the viscerocranium achieved classification accuracy of only 66.2% (Table 4). All facial subregions also provided accuracy of less than 70%. The shape of the orbital region attained the highest accuracy of 68.8%, while the maxillary region achieved the lowest overall accuracy of 60.9%. The addition of CS to the shape variables provided a substantial increase in the classification accuracy. This was valid for all landmark configurations, except the orbital region (Table 5). Thus, the accuracy achieved by the whole configuration of the viscerocranium rose up to 92.9%, demonstrating an increase of almost 27%. Based on the size and shape data, three of the facial regions (zygomatic, nasal and maxillary) achieved accuracy of more than 85%, while the orbital region showed the lowest classification result—slightly higher than 75%. Thus, the largest increase in the classification accuracy was obtained for the nasal region (nearly 23%), while that of the orbital region was only 6.8%.

## 4. Discussion

GM methods enable easy location of areas expressing the greatest degree of sexual dimorphism and indicate the specific pattern of dimorphism [69]. Based on GM methods, strong sexual dimorphism has been previously found in the viscerocranium [58] as well as in the upper face [25,56] and the orbital, nasal, and palatal region [56]. Furthermore, the upper face has been reported as the cranial region expressing the most prominent differences in shape [57]. Musilova et al. [40] have found that the facial region accumulates the most significant sex differences in the skull, although they refer mostly to its size. Thus, our results confirm the presence of significant sex differences in size and shape of the viscerocranium and its parts.

A substantial portion of the variance due to sexual dimorphism has been attributed to the cranial size [70]. Significant differences in the CS of male and female crania have been previously reported [6,25,55]. In our study, significant sex differences are detected in the size of the viscerocranium and in all of the separate facial regions. The discrimination ability of the viscerocranium size is nearly 82% in our sample, which is slightly lower than the accuracy reported by Millela et al. [58] and is higher than that obtained by Chovalopoulou and Bertsatos [57] on the upper face. Sexual dimorphism in overall facial size has been noted to develop postnatally and to increase with age up to the latest stages of development. Until puberty, the size of the viscerocranium in males is only slightly larger than that in females of the same age, but the difference becomes more pronounced at the following age stages [71]. Sexual dimorphism in facial size is clearly apparent around 14 years of age [34]. The female face stops growing around this age (14–15 years), while facial development in males continues throughout the adolescent period and into early adulthood [60,71]. Thus, the extended period of growth in males is a key moment in the development of sexual dimorphism in the facial skeleton [72]. Under normal conditions, there is a strong developmental and functional integration between growth of the human facial skeleton and its surrounding structures, such as the neural and sensory organs contained within the craniofacial skeleton and various oral, pharyngeal, and masticatory muscles [73]. However, the postnatal growth of the viscerocranium and neurocranium is influenced by epigenetic factors, showing high variability among different populations, geographical regions, socioeconomic strata, etc. [5]. In this regard, the growth of each individual advances in a specific manner and reaches a definite size modulated under the influence of multiple intrinsic and extrinsic factors.

Despite the pivotal part of the size, sexual dimorphism is also expressed in particular shape differences. These differences have a slightly different pattern in the separate population groups [74]. Specific genes are supposed to regulate the morphogenesis of the facial skeleton, and subtle differences in gene regulation amongst individuals attribute to the variation in facial skeletal morphology [73]. Nevertheless, it is still not clear what mechanisms exactly lead to these sexually dimorphic shape differences or how genetic activity is involved in shaping the phenotype [75]. During the ontogenetic development, the shape changes in the viscerocranium are considerable. The ontogenetic shape changes display an elongation of viscerocranium with the increase of age [50]. The face becomes more prognathic and there is a marked increase in relative facial height [50,53]. Sexual dimorphism in facial shape has been observed at all stages of growth [71,72], although until puberty females are more advanced in the development of facial shape than males [71].

According to the previous GM studies, the main sex difference in shape of the adult viscerocranium is observed in the width, which is relatively larger in males compared to females [20,25,55,56,58]. A sex difference has also been established in the height of the facial region, being relatively shorter in males compared to the females [56]. Franklin et al. [20] have established a flatter face in females, whereas according to Bigoni et al. [56] the females have a more convexly shaped face compared to the flatter profile among males. It has also been reported that males exhibit more projecting zygomatic bones than females [40,58]. The previous observations on the orbital region have concluded that the female orbits are relatively larger compared to the male ones [20,25]. In addition, it has been reported that the female orbits are more rounded, while the male ones are relatively shorter in height and wider, i.e., rectangular in shape [56,57,58,59]. Furthermore, it has been established that the eye sockets are placed deeper and more medially in male crania [25]. This corresponds to the results about the eye position on the human face, since it has been found that the eyes are located more laterally and anteriorly in females [76]. The previous observations on the nasal region have shown that males have relatively higher and narrower nasal aperture and more prominent nasal bones, while in females the nasal aperture is relatively wider and the nasal bones are flatter [6,56,58]. The maxillary region, and particularly its midline alveolar part, has provided some contradictory results in the previous GM studies. Bigoni et al. [56] have established that the female crania have more anteriorly projected prosthion then the male ones. The shape analysis of Musilova et al. [40] has shown that the premaxillar region in male crania is more posteriorly sloped, while the premaxilla in female crania is more vertically oriented. However, according to Green and Curnoe [55] and Millela et al. [58], the male crania are characterized by a relatively greater anterior projection of the alveolar region compared to the female ones. Although our results indicate significant shape differences in the viscerocranium of adult males and females, the inspection of shape variation in the two sexes (i.e., the PCA results) demonstrates a large overlap between the male and female crania. The latter means that a wide range of shape variants of the viscerocranium is detected in both sexes. The observed overlap could be explained with the use of size-corrected shape variables in our study. It is likely that the size-related shape variation accounts for the sex differences in cranial shape. Thus, the largest shape variance in our sample could hardly be explained by sex differences, but it originates from other factors.

In concordance with the PCA results, the shape of the viscerocranium provides low discrimination between the male and female crania in Bulgarians (only 66%). Thus, our result for the discrimination ability of the viscerocranium shape is much lower in comparison to the previous studies, reporting 83.1% in Greeks [57], 83.3% in the Czech population [25], 88.5% in Italians [58], and 100% in the Central European population [56]. Recently, we have established that the shape of the neurocranium in the same population provides comparable and even slightly lower overall accuracy (63.3%) than the viscerocranium [77]. On the other hand, the size of the neurocranium achieves higher accuracy (85.5%) than that of the viscerocranium. The combined shape and size data have provided similar accuracy rates for both neuro- and viscerocranium: 92.2% and 92.9%, respectively. Furthermore, we have explored the cranial sexual dimorphism in the Bulgarian population using machine learning algorithms [41,43] and achieved an accuracy of 92% with a set of classification rules and more than 95% applying support vector machine and artificial neural network to cranial measurements. These results confirm that the sex differences in cranial size are well expressed in the studied population, but not in cranial shape. In contrast to our results, Millela et al. [58] have established that the shape of the viscerocranium provides higher accuracy than its size (88.5% vs. 83.6%), although a slight dominance of size over shape has been observed in the whole skull (89.7% vs. 87.4%). Chovalopoulou and Bertsatos [57] have also established higher accuracy for the shape (83.1%) over size (76.5%) in the upper face, but the values were lower than those reported by Millela et al. [58].

Our results on facial subregions show that the midfacial (nasal and maxillary) region is more dimorphic in size, while the more lateral regions (orbits and zygomatic bones) are more dimorphic in shape, although the accuracy based on shape is generally rather low. According to earlier reported accuracy data, the shape of the orbits and nasal aperture has discriminated the two sexes, with an overall accuracy of less than 80% [56,57,59]. Despite the low results for the shape of the nasal region in our study, the latter demonstrates strong sex differences in its size. The higher accuracy results obtained for the size of the nasal and maxillary region could be related to the more extended growth of the nasal bones and maxilla, which reach adult size during puberty, while the orbital cavities and zygomas adopt the adult size and proportions earlier—before 10 years of age [78]. The later growth of the maxilla is associated with the replacement of deciduous teeth and eruption of the permanent teeth, mastication and increase in size of the maxillary sinus. Besides, the nasal region is related to the respiratory system and the increase of oxygen consumption in the growing organism. Furthermore, the orbital region is the least dimorphic area in size in our study and the accuracy based on its shape is only 69%. These results confirm that this region is an ineffective sex indicator, even though the shape of the orbital region achieved the highest result among the separate facial regions. The use of the craniometric approach has also shown that the orbital dimensions, in particular the orbital height, are among the least dimorphic variables and appear as unreliable parameters for sex estimation [1,7,21,22,23,33,34,35,79]. So, the early cessation of growth in the orbital region could explain the weak manifestation of sexual dimorphism in its size and shape. Regarding the zygomatic region, its size yields slightly lower accuracy than the rates for the nasal and maxillary region. Although this bone attains its adult size a bit earlier than the maxilla and nasal bones, it provides an attachment site for the masseter muscle. In this regard, the development of sex differences could be expected in attachment sites of the muscles of mastication, since mechanical loading has an impact on bone remodeling [80]. In the traditional craniometric studies, bizygomatic breadth is one of the most dimorphic metric traits [7,14,15,17,21,22,23,24,34,35,38,70]. Moreover, we have developed classification rules and decision trees for sex estimation in an earlier study, where the bizygomatic breadth or non-standard measurements including the right/left zygion are present in all of the models for sex discrimination [43]. All this comes to show that the time of growth of a bone or bone structure is important for the development of sexually dimorphic traits. However, the bone remodeling occurring after ceasing of growth could also have an impact on the manifestation of sex-specific morphological characteristics, since it is under mechanical and endocrinal influence [80].

Size has been regarded as a more accurate predictor of sex [20], since male and female skulls differ more in size than in shape [81] and the size differences are much larger than the shape ones [19]. Our study fully confirms that size is a better sex classifier than shape regarding the configuration of the viscerocranium and its separate subregions. Accordingly, the addition of CS to the shape variables considerably improves the classification accuracy. Such an increase in accuracy has been obtained in a number of previous studies [6,20,28,82]. In the form space, Bejdova et al. [25] and Millela et al. [58] have achieved accuracy rates (91–92%) similar to that obtained by us. This shows that the level of sexual dimorphism in viscerocranium based on the shape and size together is similar, but the shape and size components have a different contribution. Accordingly, Chovalopoulou and Bertsatos [57] have reported lower accuracy in the form space (86.7%). In general, the discussed population samples originated from south and east Europe and have shown similar results about the form of the viscerocranium—except for the Greeks. This result is a bit unexpected, since the Greek sample is geographically closest to that of the present study, while the Italian sample is temporally the most distant one and the Czech sample is geographically most remote compared to the other population groups. However, these results indicate that the size and shape differences should be considered together in the sex estimation process rather than the shape or size only.

Population-specificity is an important part of studies on cranial sexual dimorphism. In general, a common pattern of sexual dimorphism can be traced among the different population groups, although each population demonstrates distinct and unique morphological characteristics. Population-specificity of facial morphology develops prenatally or relatively early postnatally and endures modifications during ontogeny [52]. Basically, the initiation of the general developmental trajectory of the face commences over gestation weeks 6–9, independent of sex, and the beginning of gonadal hormone secretion after the 10th week modulates this trajectory and gives rise to sexual dimorphism in the face [76]. Thus, a possible reason for the differences in the manifestation of sexual dimorphism in various populations could be a different amount of prenatal testosterone [83]. In the postnatal period, the conditions accompanying the growth and development, and especially their longer course in males, are crucial for the evolvement of the sexual dimorphism to its full capacity. Secular changes are also an important point concerning the level of sexual dimorphism expressed in a particular population. They occur within a population under the influence of various factors and could be manifested via changes in the skeletal dimensions under altered conditions [32]. Thus, the study of sexual dimorphism on contemporary individuals is quite important in order to explore its current level. As a rule, sexual dimorphism is expressed more distinctly in populations with better living conditions and health status [56]. Therefore, a primary contributor to positive secular changes is the interaction of improved environmental quality and nutritional status [84]. Moreover, secular changes in cranial morphology occur at different rates among the populations [83]. This is an additional factor amplifying the interpopulation differences in the level of sexual dimorphism, which enhances the population-specificity of the developed sex estimation methods.

## 5. Conclusions

Our data show that the size and shape of the viscerocranium differ significantly between the male and female crania. It can be inferred that the size of viscerocranium is a more useful sex indicator in comparison to the shape. Based on size, the best accuracy is achieved by the nasal region, while the orbital region exhibits the lowest result. In general, the shape provides very low discrimination between the male and female crania. Among the facial subregions, the shape of the orbital region produces the highest accuracy, while the shape of the maxillary region provides the worst result. In the size-shape space, the full configuration of the viscerocranium achieves the highest accuracy result (93%) followed by the zygomatic region, while the orbital region yields the lowest accuracy rate.

## Figures and Tables

**Table 1:** Definition of the landmarks.

Landmarks		Description	Configurations *
1	Nasion (n)	The point of intersection of the nasofrontal suture and the midsagittal plane.	V, N
2	Rhinion (rhi)	The endpoint of the internasal suture at the lower edge of the nasal bones.	V, N
3	Subspinale (ss)	The deepest midline point below the anterior nasal spine.	V, N, M
4	Prosthion (pr)	The most anterior midpoint on the alveolar process of maxilla.	V, M
5	Frontomalare temporale (fmt)	The most lateral point on the frontozygomatic suture.	V, Z
6	Frontomalare orbitale (fmo)	The point of intersection of the zygomaticofrontal suture and the lateral orbital margin.	V, O, Z
7	Maxillofrontale (mf)	The point of intersection of the medial orbital margin and the frontomaxillary suture.	V, O, M
8	Zygomaxillare (zm)	The most inferior point on the zygomaticomaxillary suture.	V, M, Z
9	Jugale (ju)	The point corresponding to the apex of the angle between the posterior edge of the frontal process and the superior margin of the temporal process of the zygomatic bone.	V, Z
10	Zygion (zy)	The most lateral point on the zygomatic arch.	V, Z
11	Ektoconchion (ek)	The point of intersection between the lateral orbital margin and the line originating from maxillofrontale and crossing the orbit parallel to the upper orbital margin.	V, O, Z
12	Nasolaterale (nl)	The most lateral point on the margin of the piriform aperture.	V, N, M
13	Zygoorbitale (zo)	The point of intersection of the inferior orbital margin and the zygomaticomaxillary suture.	V, O, M, Z
14	Supraorbitale (so)	The most superior point on the superior orbital margin.	V, O
15	Midnasale (mn)	The deepest point on the nasal bones in the midsagittal plane.	V, N
16	Nasomaxillofrontale (nmf)	The point located on the junction of the frontal, maxillary and nasal bones.	V, N, M
17	Nasomaxillare (nm)	The most inferior point of the nasomaxillary suture at the intersection with the piriform aperture.	V, N, M
18	Infraorbitale (io)	The most superior point on the margin of the infraorbital foramen.	V, M
* Configurations in which the corresponding landmark is included: V–viscerocranium; O–orbital region; N–nasal region; M–maxillary region; Z–zygomatic region. Landmark definition: 1–10–Martin and Saller [62]; 11–12–Alekseev and Debets [63]; 13–Howells [64]; 14–18–not traditionally defined craniometric landmarks.			

**Table 2:** Centroid size (in mm) of the configurations in male and female crania.

Landmark Configuration	Males		Females		t-Test/U-Test /*p*-Value/
	Mean	SD	Mean	SD	
Viscerocranium	249.52	8.32	235.10	6.91	U = 2653.00, *p* ≤ 0.001
Orbital region	125.00	5.02	120.62	4.17	t = 8.779, *p* ≤ 0.001
Nasal region	67.25	3.33	61.98	2.78	U = 3083.00, *p* ≤ 0.001
Maxillary region	126.59	5.04	118.51	3.93	U = 3118.00, *p* ≤ 0.001
Zygomatic region	208.67	7.50	197.16	6.26	U = 3426.00, *p* ≤ 0.001

**Table 3:** Classification accuracy (in %) based on the centroid size.

Landmark Configuration	Males	Females	Total
Viscerocranium	80.8	82.6	81.8
Orbital region	70.5	69.6	70.0
Nasal region	79.5	84.8	82.4
Maxillary region	77.6	84.2	81.2
Zygomatic region	78.2	80.4	79.4

**Table 4:** Classification accuracy (in %) based on the shape variables.

Landmark Configuration	Males	Females	Total
Viscerocranium (PC1-30)	66.0	66.3	66.2
Orbital region (PC1-9)	69.9	67.9	68.8
Nasal region (PC1-10)	63.5	65.2	64.4
Maxillary region (PC1-16)	59.0	62.5	60.9
Zygomatic region (PC1-12)	66.0	65.8	65.9

**Table 5:** Classification accuracy (in %) based on the combined data of size and shape.

Landmark Configuration	Males	Females	Total
Viscerocranium (PC1-27)	92.9	92.9	92.9
Orbital region (PC1-9)	71.8	78.8	75.6
Nasal region (PC1-10)	87.8	86.4	87.1
Maxillary region (PC1-16)	82.1	89.1	85.9
Zygomatic region (PC1-11)	86.5	89.7	88.2

## Data Availability

The data presented in the study are available on request from the corresponding author.

## Supplementary Materials

The following supporting information can be downloaded at: https://www.mdpi.com/article/10.3390/biology11091333/s1, Table S1. Intraobserver measurement error of the landmarks /in mm/.
