# Supplementary material for: A Geometric Morphometric Study on Sexual Dimorphism in Viscerocranium
Source: Biology (Basel). 2022 Sep 9;11(9):1333. doi: 10.3390/biology11091333 (PMC9495862; doi:10.3390/biology11091333)
Supplement: Supplementary file 1 [file biology-11-01333-s001.zip › biology-1890791-supplementary.pdf]

Table S1. Intraobserver measurement error of the landmarks /in mm/.

Landmark	Intraobserver error
Nasion	0.25
Rhinion	0.18
Subspinale	0.28
Prosthion	0.22
Frontomalare temporale (right)	0.27
Frontomalare temporale (left)	0.28
Frontomalare orbitale (right)	0.18
Frontomalare orbitale (left)	0.18
Maxillofrontale (right)	0.47
Maxillofrontale (left)	0.44
Zygomaxillare (right)	0.35
Zygomaxillare (left)	0.33
Jugale (right)	0.26
Jugale (left)	0.27
Zygion (right)	0.53
Zygion (left)	0.47
Ektoconchion (right)	0.46
Ektoconchion (left)	0.50
Nasolaterale (right)	0.27
Nasolaterale (left)	0.29
Zygoorbitale (right)	0.39
Zygoorbitale (left)	0.43
Supraorbitale (right)	0.51
Supraorbitale (left)	0.51
Midnasale	0.22
Nasomaxillofrontale (right)	0.23
Nasomaxillofrontale (left)	0.22
Nasomaxillare (right)	0.19
Nasomaxillare (left)	0.18
Infraorbitale (right)	0.16
Infraorbitale (left)	0.15
